# Examining the Influence of Modifiable Risk Factors for ADRD in a Rural Cohort

**DOI:** 10.1093/geroni/igaf122.022

**Published:** 2025-12-31

**Authors:** Christine Williams, Lisa Wiese, Janet Holt, Juyoung Park

**Affiliations:** Florida Atlantic University, Boca Raton, Florida, United States; Florida Atlantic University, Boca Raton, Florida, United States; Florida Atlantic University, Boca Raton, Florida, United States; University of Arizona, Tucson, Arizona, United States

## Abstract

Results from year one of a three-year repeated measures descriptive study involving multicultural rural residents are offered (N = 494). The region surrounding Lake Okeechobee, 2nd largest freshwater lake in the contiguous U.S., is home to diverse groups, including farmworkers, teachers, and healthcare workers who experience all 14 potentially modifiable risk factors for Alzheimer’s Disease and Related Dementias (ADRD). We report on these risk factors (Livingston et al., 2023). For example, correlations between individual risk factors and cognitive function (Montreal Cognitive Assessment) scores revealed that education, r =.16, p < .001, hypertension, r = -.12, p = .008, hearing loss, r = -.14, p = .002, smoking, r = -.13, p = .005, physical performance, r = .22, p < .001, cholesterol, r = -.10, p = .02, depression, r = -.15, p < .001, loneliness, r = -.13, p = .003, and social connectedness, r = .22, p < .001, correlated with MoCA scores. Number of years lived in this area interacted with education, p = .007, and diabetes, p = .038, to predict cognitive function. Interactions with smoking, BMI, social connectedness, and loneliness were notable trends towards significance (p < .1). This highlights the need to examine all ADRD risk factors prior to developing community-based interventions to decrease risk, while considering that older age correlates with many other risk factors for ADRD. Future studies will focus on improving knowledge about ADRD, health behaviors, early detection of cognitive decline and management of chronic conditions related to ADRD risk.

